# Characterization of FANCL variants observed in patient cancer cells

**DOI:** 10.1042/BSR20191304

**Published:** 2020-06-04

**Authors:** Mark G. Frost, Amir Mahdi Mazloumi Aboukheili, Rachel Toth, Helen Walden

**Affiliations:** 1 MRC Protein Phosphorylation and Ubiquitylation Unit, Sir James Black Centre, School of Life Sciences, University of Dundee, Dundee DD1 5EH, U.K.; 2 Institute of Molecular Cell and Systems Biology, University of Glasgow, Glasgow G12 8QQ, U.K.

**Keywords:** DNA synthesis and repair, Fanconi Anemia, mutation

## Abstract

Fanconi Anemia (FA) is a rare genetic disorder characterized by developmental defects, bone marrow failure and high predisposition to cancer. The FA DNA repair pathway is required in humans to coordinate repair of DNA interstrand cross-links. The central event in the activation of the pathway is the monoubiquitination of FANCD2 and FANCI by the E2-E3 pair, Ube2T-FANCL, with the central UBC-RWD (URD) domain of FANCL recognizing the substrates. Whole genome sequencing studies of cancer cells from patients identified point mutations in the FANCL URD domain. We analysed 17 such variants of FANCL, including known substrate binding mutants (W212A, W214A and L248A, F252A, L254A, I265A), a FA mutation (R221C) and 14 cancer-associated mutations (F110S, I136V, L149V, L154S, A192G, E215Q, E217K, R221W, T224K, M247V, F252L, N270K, V287G, E289Q) through recombinant expression analysis, thermal shift assay, interaction with FANCD2, *in vitro* ubiquitination activity, and cellular sensitivity to an interstrand cross-linking agent. We find that the FANCL mutations I136V, L154S, W212A and L214A, R221W, R221C, and V287G are destabilizing, with N270K and E289Q destabilizing the C-terminal helices of the URD domain. The hydrophobic patch mutant (L248A, F252A, L254A, I265A), along with mutations E217K, T224K, and M247V, cause defects in the catalytic function of FANCL. This highlights the C-terminal lobe of the FANCL URD domain as important for the activity and function of FANCL. These mutations which affect the fold and activity of FANCL may contribute to tumorigenesis in these non-FA cancer patients, and this implicates FA genes in general cancer progression.

## Introduction

**Figure 1:**
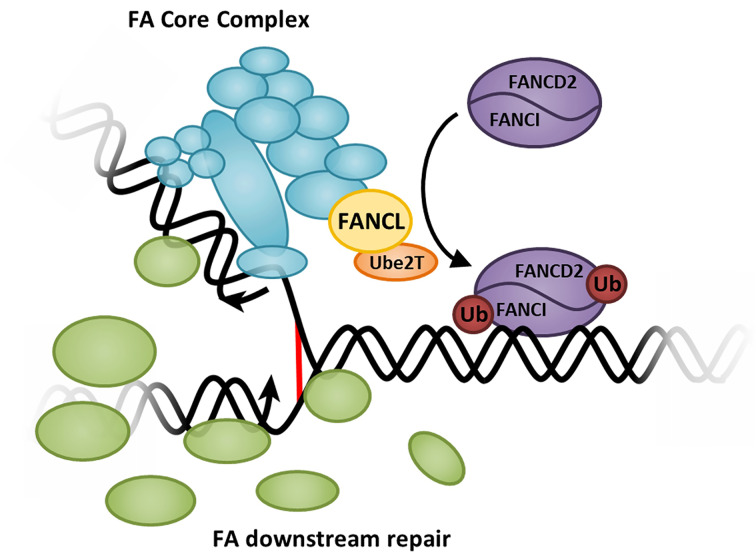
The Fanconi Anemia DNA repair pathway A covalent interstrand cross-link (ICL) (red) results in a stalled replication fork, which is recognized by the FA core complex (cyan), including the subunit FANCL (yellow) that has E3 Ubiquitin ligase activity. FANCL pairs with the E2 conjugating enzyme, Ube2T (orange), and together they monoubiquitinate FANCD2 and FANCI (purple), resulting in their localization to the DNA, and recruitment of downstream repair factors (green) that coordinate resolution of the lesion and the stalled replication fork.

Fanconi anemia (FA) is a rare genetic disorder characterized by skeletal and developmental defects, bone marrow failure and high predisposition to cancer [1]. Defects in the Fanconi Anemia DNA Repair Pathway lead to an inability to repair covalent DNA interstrand cross-links (ICLs) [2], leading to inhibition of transcription and translation, and chromosomal instability. Currently, there are 21 genes known to be mutated in cases of FA [3]. Of these genes, nine comprise a multiprotein core complex that can monoubiquitinate FANCD2 and FANCI, the FA pathway’s substrates (Figure 1). The other FA genes are involved either upstream or downstream of this event. Ube2T and FANCL are the cognate E2-E3 pair that are able to monoubiquitinate the substrate proteins [4,5], and this is the critical event for activation of the pathway. Mutations in any of the 9 FA genes that form the core complex (CC) lead to a defect in substrate monoubiquitination; however, *in vitro* Ube2T and FANCL are sufficient to monoubiquitinate FANCD2 or FANCI [4,6,7]. Additionally, invertebrates appear to have a reduced complement of FA genes, such as *Drosophila melanogaster* that has homologues of only FANCM, Ube2T, FANCL, FANCD2 and FANCI [8]. This suggests that key activity for the selective monoubiquitination of FANCD2 and FANCI are achieved by Ube2T and FANCL, with a recent *in vitro* study demonstrating FANCL can allosterically activate Ube2T to support site-specific FANCD2 ubiquitination [9].

FA patients are highly predisposed to cancer, indicating that defects in the pathway can contribute to cancer progression, due to decreased chromosomal stability and increased mutation rates. Interestingly, some mutations in FA genes, such as FANCL, which have been observed in patients’ cancer cells have not been found in FA patients [10,11]. For example, in one study 3.8% of patient samples were found to have missense mutations in FANCL [12] across various cancer types. The severity of effect of these mutations in FANCL are unknown, and could potentially give an insight into the function of FANCL. Therefore we sought to characterise the effect of these mutations.

The structure of FANCL reveals three distinct domains: an N-terminal E2-like fold (ELF) domain, a central double RWD fold (DRWD), or UBC-RWD (URD) domain in human FANCL, and a C-terminal RING domain [13,14]. The ELF domain has been shown to bind to ubiquitin [15], and recent evidence suggests this domain binds to FANCE [16], the RING domain binds to Ube2T [17] as well as the substrate, FANCI [16], and the central URD domain is able to bind to the substrates, FANCD2 and FANCI [14], though the details of this interaction are only recently coming to light [16,18]. The present study focuses on mutations in the URD domain of FANCL, to determine whether these mutations affect FANCL’s recognition of the substrates or ubiquitination activity.

**Figure 2:**
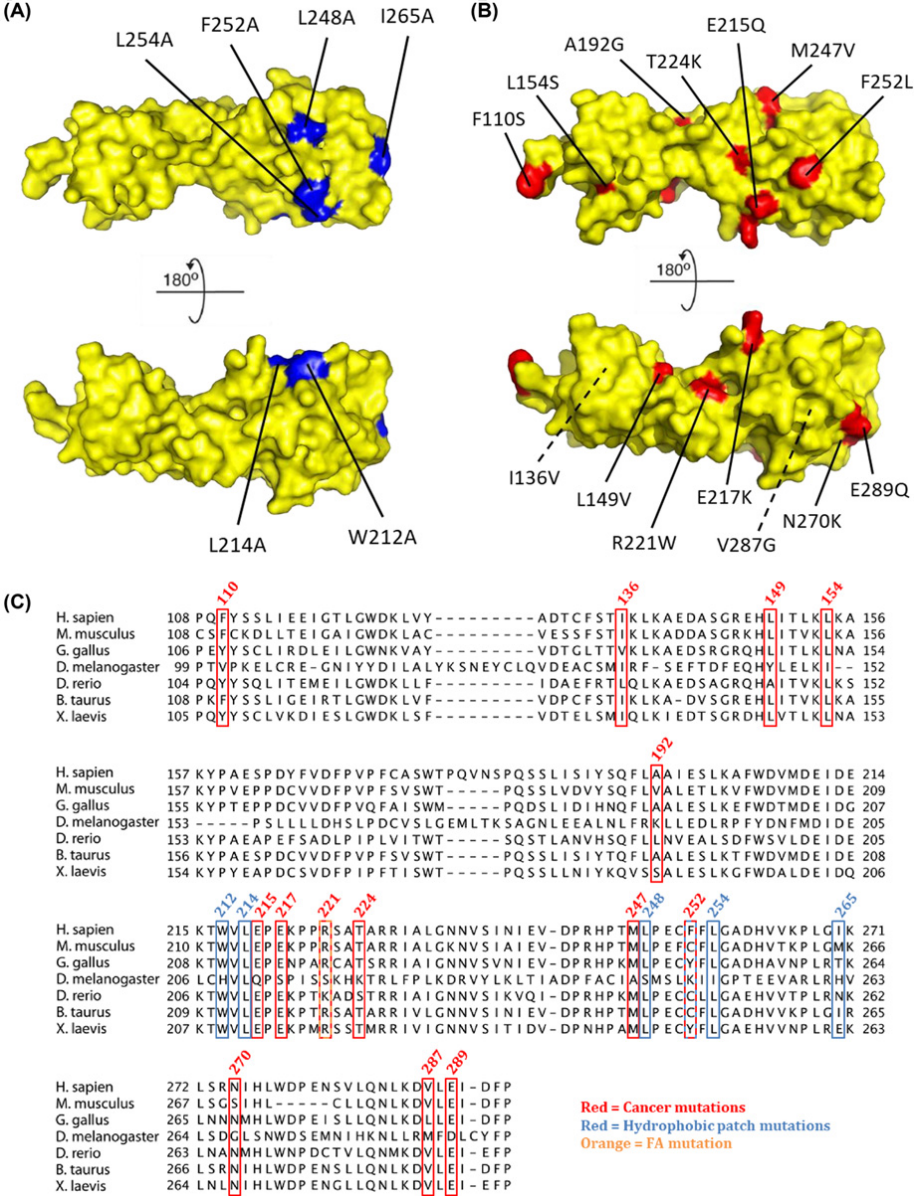
FANCL URD domain mutations The surface representation crystal structure of (**A**) FANCL URD (PDB: 3ZQS) (yellow) with FANCL hydrophobic patch mutants known to decrease FANCD2 and FANCI binding [14] highlighted in blue, and (**B**) FANCL URD with cancer mutations highlighted in red. (**C**) FANCL URD sequence alignment. The sequences of FANCL from the species indicated were aligned using MafftWS, and the figure generated by JalView. Mutations found in cancer cells are highlighted in red, hydrophobic patch mutations [14] are highlighted in blue, and the FA mutation is highlighted in orange.

Here, we characterize FANCL URD hydrophobic patch mutations that have previously been shown to impair substrate binding [14] (Figure 2A), the FA mutation R221C [19], and mutants found from cancer cell sequencing studies collated in cBioPortal (Figure 2B) [10,11]: F110S [20], I136V [12], L149V [21], L154S, A192G [22], E215Q [23], E217K [24], R221W [25], T224K, M247V, F252L, N270K, V287G [25], E289Q [26]. We characterize the effect of these FANCL mutations on protein stability (and aggregation) during expression, a thermal shift assay, their binding affinity for FANCD2, their *in vitro* FANCD2 monoubiquitination activity, and their effect on cellular sensitivity to the ICL agent MMC.

We find that the FANCL cancer-associated mutations I136V, L154S, W212A and L214A, R221W, R221C, and V287G destabilize the FANCL protein by disrupting the protein fold. Likewise, the mutations N270K and E298Q, which are in the final two C-terminal helices of the central FANCL URD domain, result in FANCL protein destabilization. We also found that a FANCL hydrophobic patch mutant known to decrease FANCD2 and FANCI binding (L248A, F252A, L254A, I265A) as well as mutations E217K, T224K and M247V caused defects in the function of FANCL, both in *in vitro* ubiquitination activity and in cells increasing sensitivity to interstrand-crosslinking agents. The cancer-associated mutations that cause a defect in the function of FANCL could have contributed to the progression of cancer in patients, and highlights residues important for the function of FANCL.

## Methods

### Site-directed mutagenesis

Mutations were made in the FANCL gene using the QuickChange® Site-Directed Mutagenesis (Stratagene) method. The template plasmid contained codon optimised (GeneArt) Human FANCL URD-RING (residues 109–375) coding sequence and an N-terminal His-Smt3 tag, as previously described [14]. Primers were designed with the desired nucleotide changes, then the template plasmid was replicated using these primers with KOD Hot Start DNA Polymerase (Sigma Aldrich) as per the manufacturer's instructions. Template DNA was then digested using Dpn1 (NEB). Mutagenesis was confirmed by sequencing.

### Protein expression and purification

We transformed *Escherichia coli* BL21 cells (Invitrogen) with plasmids containing either Ube2T or the variants of FANCL URD-RING domains. We transferred these cells to lysogeny broth medium supplemented with antibiotics and with 0.5 mM ZnCl_2_. Cells were cultured at 37°C to an *A*_600nm_ of 0.6, induced with 0.25 mM isopropyl-1-thio-β-d-galactopyranoside, and cultured overnight at 16°C. Cells were harvested by centrifugation and lysed by sonication on ice (10 s bursts followed by 30 s on ice, repeated six times) in 100 mM Tris, pH 8.0, 500 mM NaCl, 20 mM imidazole, 250 µM tris(carboxyethyl)phosphine. Cell lysate was centrifuged at 35,000 × ***g*** at 4°C to remove cell debris. Clarified supernatants were applied to a Ni^2+^-nitrilotriacetic acid (Ni-NTA) affinity resin (Invitrogen) equilibrated with lysis buffer. The 6xHis-Smt3 tag was removed overnight at 4°C with Smt3 protease, Ulp1, at a w/w of 1:30 Ulp1:His-Smt3-protein. The protein was eluted from the Ni-NTA with 100 mM Tris, pH 8.0, 100 mM NaCl, 10% glycerol, 250 µM tris(carboxyethyl)phosphine. FANCL protein was applied to a MonoQ anion exchange column. The first peak eluted from a NaCl gradient was collected and concentrated. All proteins were applied to a Superdex 75 column for size-exclusion chromatography. Purified proteins were concentrated to ∼3 mg/ml and flash-frozen in liquid nitrogen, and stored at −80°C. *Xenopus laevis* FANCD2 was expressed and purified as previously described [27]. Briefly, *Sf21* cells were infected with a baculovirus containing the FANCD2 gene and incubated for 72 h. Cells were pelleted at 1000 × ***g*** for 5 min, and then lysed by sonication at 35% amplitude for 8 pulses of 10 s. Cell lysate was clarified by centrifugation as above, and purified with Ni-NTA as above. After elution from Ni-NTA the protein was loaded onto a Heparin column (GE Healthcare), then eluted with a NaCl gradient. The protein was then further purified by size exclusion with a Superose 6 16/600 column (GE Healthcare). Protein was concentrated, flash frozen and stored at −80°C.

### *In vitro* pulldown assay

For each mutant of FANCL URD-RING 10 µg of recombinant protein was incubated with 0.5 µg of 6xHis-FANCD2 and magnetic Ni-NTA (Invitrogen). The proteins were mixed in the assay buffer: 100 mM Tris pH 8, 100 mM NaCl, 0.05% Triton-X, 0.1% BSA and incubated with end-over-end agitation at 4°C. After incubation, the beads were washed three times with 500 µl of assay buffer. The beads were then boiled with LDS loading buffer, boiled, and run on SDS-PAGE, stained with Instant Blue (Expedeon), then scanned with a LI-COR system and quantified using Odyssey software.

### Thermal shift assay

Protein unfolding in response to a temperature gradient was monitored by SYPRO Orange fluorescence. The thermal shift experiments were recorded on a Bioradicycler iQ5 RT-PCR machine (Bio-rad). 2.5× SYPRO Orange dye was mixed with 3 µg of protein and diluted to a final volume of 50 µl in an opaque 96-well PCR plate (Bio-rad). The temperature was ramped from 10°C to 85°C in 0.5°C increments, and fluorescence was measured in Channel 2 (excitation 535 nm, emission 556 nm). The first derivative was used to determine the melting temperature of each sample.

### FANCD2 monoubiquitination assay

The ability of FANCL with Ube2T to monoubiquitinate FANCD2 was assessed by an *in vitro* monoubiquitination assay. Ubiquitin was fluorescently labelled with DyLight™ 800 Maleimide (Life Technologies, 46621). Human ubiquitin (2-76) was expressed with an additional N-terminal GPLCGC peptide sequence. The cysteine residue in this peptide sequence was targeted with DyLight™ 800 Maleimide according to the manufacture’s protocols. Labelled Ubiquitin was then purified by cation exchange chromatography and stored at −20°C. Reaction volumes of 10 µl contained 25 nM His-UBE1, 0.1 µM Ube2T, 0.2 µM FANCL URD-RING, 2 µM Ubiquitin^IR800^, 2 µM *Xenopus laevis* FANCD2, and reaction buffer: 25 mM HEPES (pH 8), 75 mM NaCl_2_, 1 mM MgCl_2_, 2.5 mM DTT, 1% Glycerol and 2 mM ATP. Reactions were incubated for 10 min at 30°C A 10 µl volume of LDS buffer (Invitrogen) containing β-ME was used to terminate reactions. Samples were boiled and loaded onto a 4–12% SDS-PAGE gel. Labelled ubiquitin was detected by fluorescence scanning on a LI-COR system. The fluorescence intensity of the bands corresponding to monoubiquitinated FANCD2 were quantified in Odyssey (LI-COR) software.

### Cell culture and CRISPR-Cas9

U2OS Flp-In T-REx cells were cultured in 10 cm dishes in DMEM (Dulbecco’s modified Eagle’s medium; Life Technologies) supplemented with 10% (v/v) foetal bovine serum, 2 mM L-glutamine, 100 units/ml penicillin and 0.1 mg/ml streptomycin.

FANCL CRISPR-Cas9 knockout cell lines were obtained as previously described [28]. Guide RNAs were designed to target exon 1 of FANCL (sense: GCGTGTATGAGGGATTCATCT; anti-sense: GCAGGGGGCACTGGCGCAAC). Cells were transfected with plasmids containing the gRNA and the Cas9 D10A nuclease using Genejuice transfection reagent (EMD Millipore). Cells were then selected with antibiotics, and sorted to single cells in polylysine coated plates. Surviving colonies were then screened by Western blot for FANCL protein levels.

Cells were lysed in lysis buffer (50 mM HEPES pH 7.5, 150 mM NaCl, 270 mM Sucrose, 1% Triton-X, 0.5% NP40), briefly sonicated, and incubated on ice with 50 Units Benzonase nuclease (Sigma Aldrich) for 20 min. Cell debris was cleared by centrifugation at 17,000 × ***g*** for 10 min, and the cell lysates were flash frozen in liquid nitrogen and stored at −80°C.

### Western blotting

Protein was transferred from a SDS-PAGE gel to a Nitrocellulose membrane using an iBlot transfer system (Life Technologies) at 20 V for 7 min. Membranes were blocked with 10% Milk in TBS-tween buffer (20 mM Tris pH 7.6, 150 mM NaCl, 0.1% Tween) for at least 10 min. After three 5-min washes with TBS-tween buffer, the membranes were incubated with primary antibody (in TBS-tween plus 3% BSA) overnight at 4°C. The membranes were then washed with TBS-tween and incubated with the appropriate fluorescently labelled secondary antibody at a dilution of 1:10,000 for use with the LI-COR fluorescent imaging system. Membranes were washed and scanned for fluorescence. LI-COR scans were analysed and quantified using LI-COR Odyssey software.

### Fluorescence-activated cell sorting

Cells were seeded at low confluency in 10-cm dishes. For induction of Flp-In genes, the cells were treated with Tetracyclin (varying concentration), and all cells were incubated for 24 h before treatment with 7.5 ng/ml MMC for 48 h. Cells were harvested, pelleted and washed once in PBS before fixing in ice-cold 70% ethanol, then stained with propidium iodide. Cell cycle distribution was analysed using a FACS Canto machine, and Flo Jo software. A total of four replicates were collected.

## Results

### Expression and purification of WT and mutants of FANCL

**Figure 3:**
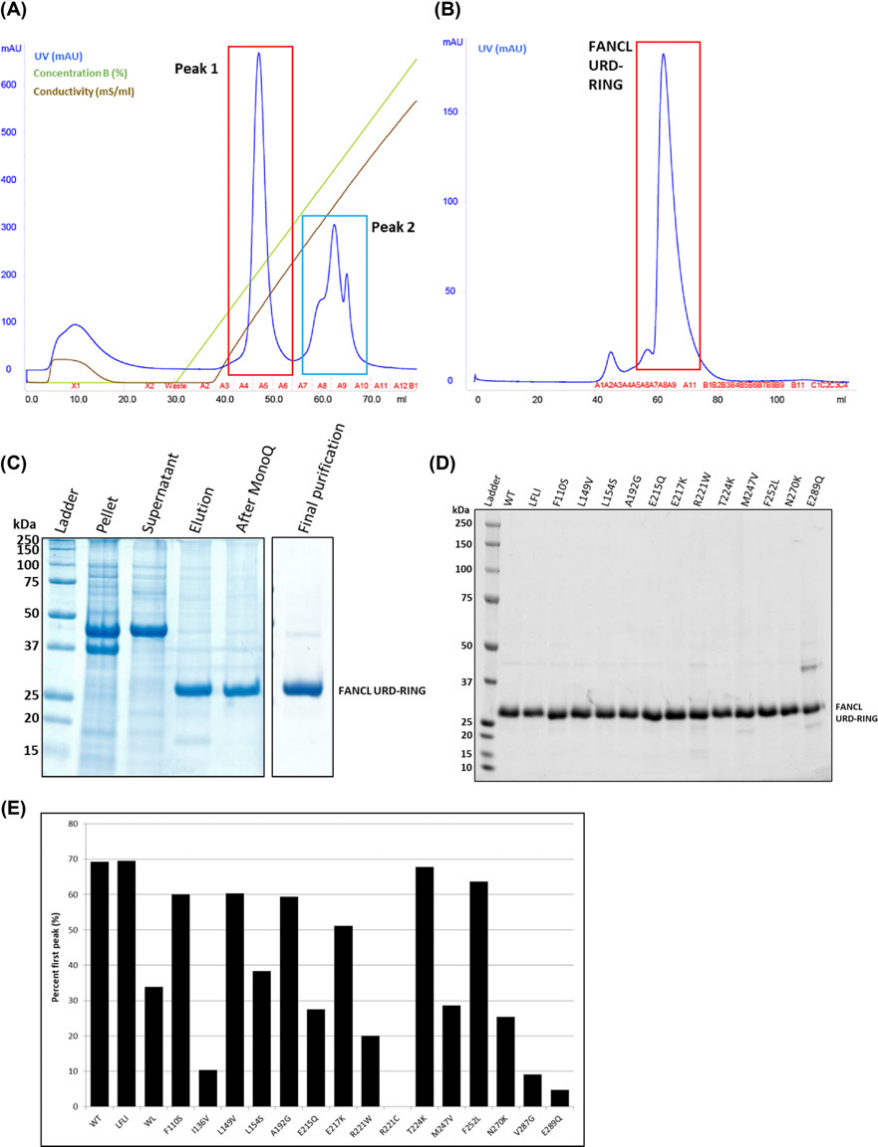
FANCL recombinant expression and purification (**A**) FANCL protein eluted with NaCl from a Mono Q anion exchange column eluted in two peaks, with peak 2 (highlighted in blue) containing improperly folded protein aggregates that elute at the void volume, whereas peak 1 (highlighted in red) contains properly folded protein, which (**B**) when run on a size exclusion chromatography column resolved the protein at the expected volume of a monomer. (**C**) An example coomassie-stained SDS-PAGE gel showing the purification of FANCL URD-RING from *E. coli* cells. (**D**) A coomassie-stained SDS-PAGE gel showing the final purified variants of FANCL URD-RING. (**E**) The percentage of protein in the first peak was calculated as an estimate based on the UV chromatogram peak heights, and this indicates the effect of FANCL mutations on the stability and fold of the expressed protein.

*Homo sapiens* FANCL URD-RING was expressed and purified by His-tag purification followed by anion exchange chromatography and size exclusion chromatography. The anion exchange column elution profile shows separation of FANCL into two species, as indicated by the two peaks (Figure 3A). The first peak contains well folded protein, resolving at the expected molecular weight position during size exclusion chromatography as a single, symmetrical peak (Figure 3B,C). The second peak eluting from the anion exchange column, while containing FANCL protein, subsequently elutes from a size exclusion column in the void volume, suggesting the protein forms large protein aggregates without monomeric well folded protein. Final, highly purified, FANCL URD-RING protein concentration was quantified by band intensity on a Coomassie-stained gel (Figure 3D). Interestingly, the ratio between the two peaks from the anion exchange chromatography differs for each mutant (Figure 3E). The ratio between these two peaks, for each mutant, is therefore an indication of the effect of the mutations on the stability and expression of FANCL.

**Figure 4:**
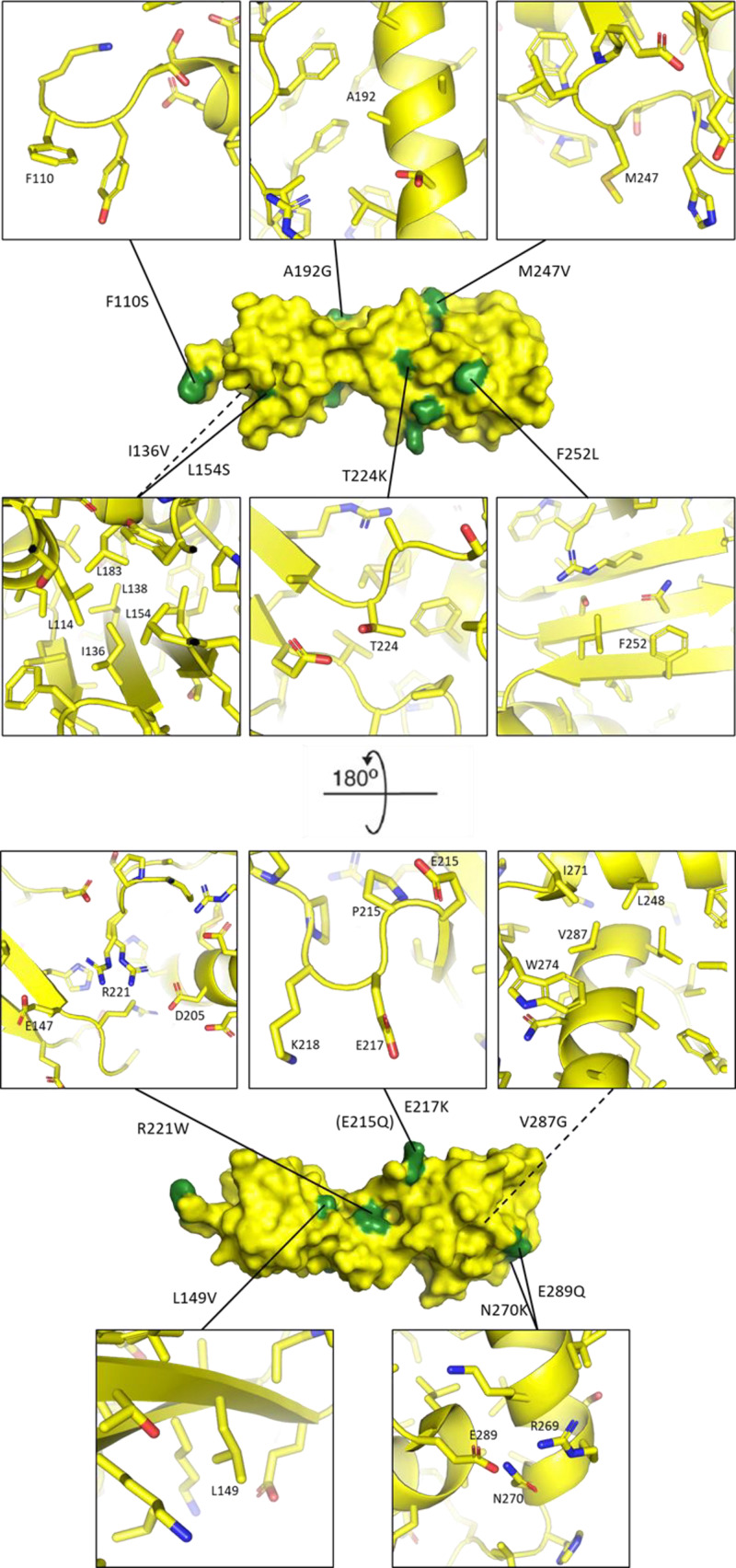
Atomic environment of FANCL residues which are mutated Two rotated views of the crystal structure of the FANCL URD domain (PDB: 3ZQS) are shown, with mutated residues highlighted in green. The immediate environment of individual residues is shown, with Carbon atoms coloured yellow, Oxygen atoms coloured red, and Nitrogen atoms coloured blue. Residues of interest are labelled.

The WT FANCL is the most stable, which is expected as there are no mutations to potentially disrupt the structure, and this is what is normally expressed in healthy cells. The cancer mutation R221W causes a 3.5-fold decrease in the amount of protein in the well-folded peak, while with this residue mutated to cysteine (R221C), as found in a Fanconi Anemia patient, there is a complete loss of properly folded protein (Figure 3E). The mutants I136V and V287G are mutations of internal hydrophobic residues (Figure 4), and results in almost all protein produced being insoluble. A small amount of protein appears entirely in the second peak after anion exchange chromatography, further highlighting the instability of the protein. A similar observation was made for the W212A, L214A surface hydrophobic patch mutation, which has previously been shown to inhibit substrate binding [14]. In fact the I136V, W212A and L214A, R221C, and V287G mutations result in such a small amount of soluble protein that no further characterization is possible.

L154 is an internal hydrophobic residue (Figure 4), and the mutation of this residue results in less stable protein, as there is a reduction in the amount of protein in the first peak by 45% compared with wild-type. E215 is a solvent exposed residue (Figure 4), yet its mutation also affects protein stability. The amount of protein in the first peak is equally decreased for the mutation of residue M247, which is on a loop of the C-terminal lobe, and is adjacent to other hydrophobic residues (Figure 4), which may structurally stabilise the wild-type protein. N270 and E289 are at the C terminus of the URD domain, and possibly interact with each other (Figure 4) or other nearby residues to stabilize the final loop and α helix of the domain.

The FANCL mutants (which expressed sufficient soluble protein for further characterisation) were purified to a high degree of homogeneity, although the less stable mutants (R221W and E289Q) had a higher proportion of impurities, due to a lower yield of FANCL URD-RING. Protein concentration was calculated from band intensity from the Coomassie-stained gel in order to exclude impurities from the concentration calculations (Figure 3D).

### Thermal unfolding of WT and the mutants of FANCL

**Figure 5:**
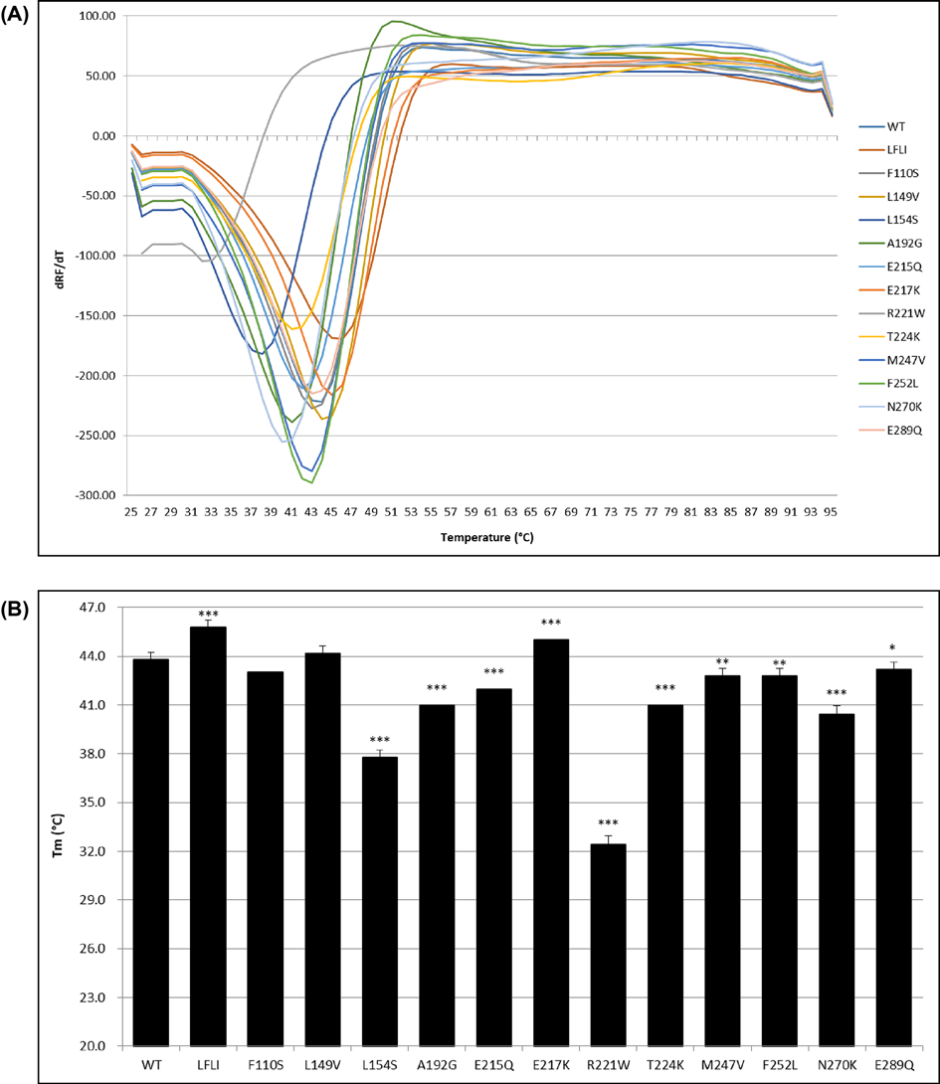
Thermal shift assay of FANCL mutants FANCL protein was mixed with SYPRO orange dye, and dispensed into a plate with five replicates for each mutant. The temperature was increased from 25°C to 95°C in 1°C increments, measuring the fluorescence signal at each interval. (**A**) The first differential of the fluorescence signal was calculated, and the apex of this curve taken to be the melting temperature. (**B**) The melting temperatures are shown for each FANCL variant. The mean and SEM were calculated from the five replicates. Statistical significance calculated by *t-*test is shown for *P*<0.1 (*), *P*<0.01 (**) and *P*<0.001 (***).

In order to further characterize the effect of these mutations on protein stability, the thermal denaturation melting temperatures were determined using a thermal shift assay (Figure 5). Only F110S and L149V do not result in a change in the melting temperature from the 43.8°C for WT FANCL URD-RING. The mutations that have the largest effect on the melting temperature of FANCL are L154S (−6.0°C) and R221W (−11.4°C), suggesting that these mutations most destabilize the FANCL URD domain.

### The effect of FANCL mutations on its interaction with FANCD2

**Figure 6:**
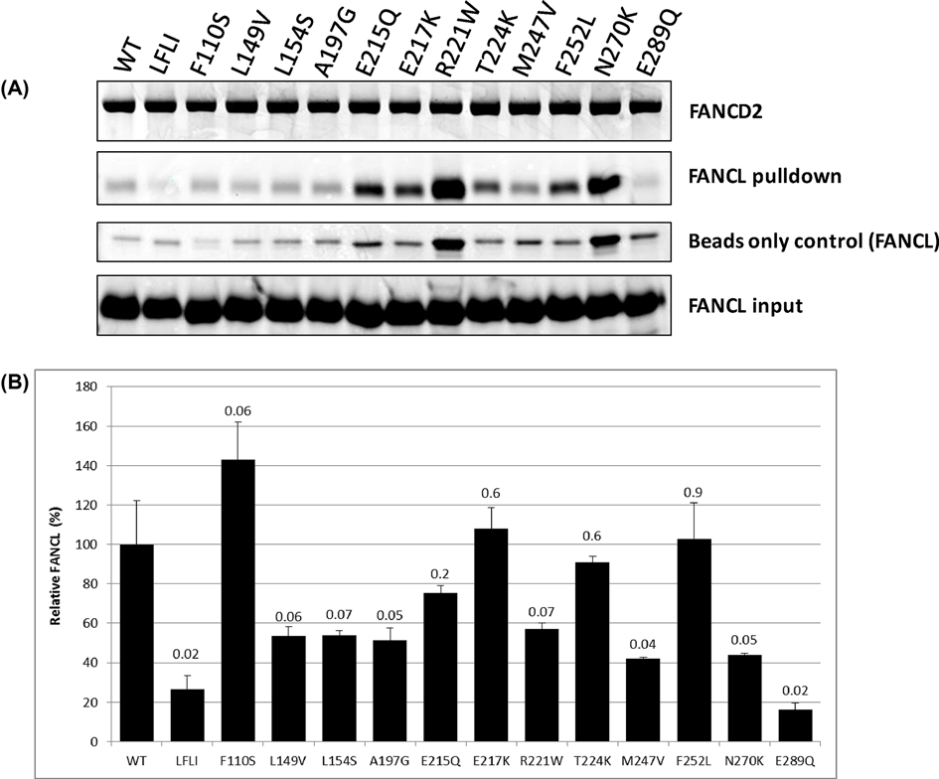
FANCD2 pulldown of FANCL mutants (**A**) Each variant of FANCL was incubated with FANCD2 and magnetic Ni-NTA at 4°C for 2 h in the assay buffer: 100 mM Tris pH 8, 100 mM NaCl, 0.05% Triton-X, 0.1% BSA. FANCL was also incubated with beads not loaded with FANCD2 to provide a control for non-specific binding. After washing with 500 μl of buffer three times, the beads were boiled with LDS loading buffer, run on SDS-PAGE, and Coomassie stained. (**B**) The gels were scanned and quantified, with the amount of FANCL adjusted for non-specific binding (beads only control), and normalized to WT FANCL.

The FANCL URD domain has been shown to bind to FANCD2 and FANCI [14], it is possible that the mutations disrupt the interaction with the substrates, causing a defect in the function of FANCL. The binding of WT and the mutants of FANCL to FANCD2 was assessed with an *in vitro* pulldown assay with 6xHis-tagged FANCD2 as the bait, and the different FANCL proteins as the prey (Figure 6). There is a pattern of non-specific binding of FANCL to the Ni-NTA beads which matches the instability profiles, so may be due to aggregation and accumulation of the FANCL with the beads. The amount of non-specific binding was corrected for by subtracting the levels of non-specific bound FANCL from the amount with FANCD2 present (Figure 6B). The hydrophobic patch mutation that has previously been shown to reduce substrate binding [14] (L248A, F252A, L254A, I265A), A192G, M247V, N270K and E289Q all show a decreased level of binding to FANCD2 compared with wild-type.

The level of substrate binding is decreased for M247V, N270K and E289Q, which increased the proportion of insoluble protein during recombinant expression, and decreased melting temperatures. It is possible that these mutants’ destabilizing of the fold of FANCL compromises their ability to bind to FANCD2, rather than these residues being directly involved in substrate binding.

### *In vitro* ubiquitination activity of WT and mutants of FANCL

**Figure 7:**
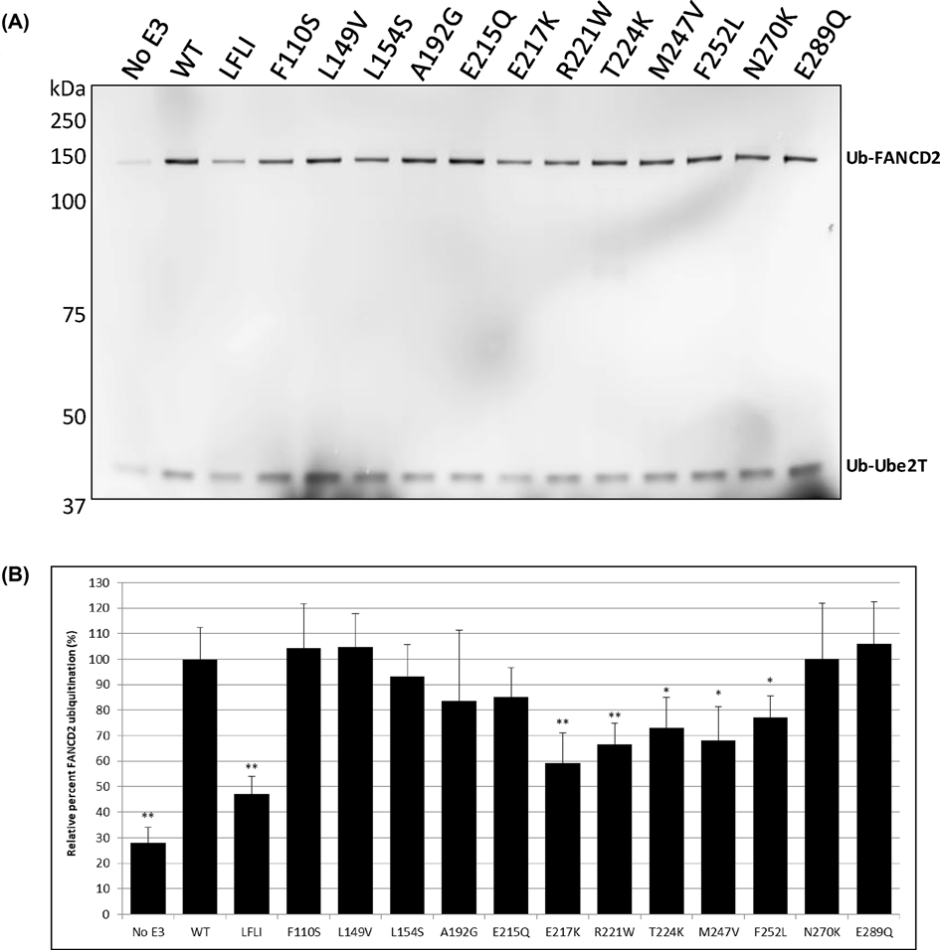
*In vitro* FANCD2 ubiquitination assay (**A**) The FANCL variants were assayed for FANCD2 ubiquitination activity *in vitro* by mixing with Ube1, Ube2T, fluorescently labelled Ubiquitin, in an assay buffer containing ATP. After 10-min incubation at 30°C, the reaction was boiled with a reducing LDS loading buffer, run on SDS-PAGE and the gel was scanned for florescence by LI-COR. (**B**) The bands corresponding to ubiquitinated FANCD2 were quantified and the mean results of eight replicates are shown with standard error of the mean indicated. Statistical significance calculated by ttest is shown for *P*<0.05 (*) and *P*<0.005 (**). The FANCL mutants E217K, R221W, T224K, M247V and F252L show a decrease in FANCD2 ubiquitination compared with wild-type.

FANCL is the subunit of the FA core complex possessing E3 ubiquitin ligase activity, and together with the E2 enzyme, Ube2T, transfers a single ubiquitin moiety to FANCD2. In order to assess the effect of mutations on the enzymatic activity of FANCL, we used an *in vitro* ubiquitination assay for the monoubiquitination of the substrate FANCD2 (Figure 7). The wild-type FANCL stimulates ubiquitination of FANCD2 by 3.6-fold compared with Ube2T alone. The FANCL hydrophobic patch mutant known to decrease FANCD2 and FANCI binding (L248A, F252A, L254A, I265A) reduces FANCD2 ubiquitination to 47% of WT (Figure 7B), with the largest effect of the FANCL mutations screened. The mutants E217K, R221W, T224K, M247V and F252L also significantly decreased FANCL’s ubiquitination of FANCD2. These residues are all contained in the C-terminal lobe of the URD domain, on the same surface (Figure 2B), which indicates that this region could be involved in the binding to FANCD2 and the catalytic activity of FANCL.

### The effect of FANCL mutations on cellular sensitivity to DNA cross-linking

Defects in the FA pathway lead to a cellular sensitivity to DNA interstrand cross-linking agents. One hallmark of this defect is cell cycle arrest at G2, as the cells are unable to resolve the cross-links. A cell cycle assay of cells with WT and the mutant forms of FANCL was used to assess the effect of the mutations on the ability of cells to overcome ICLs.

**Figure 8:**
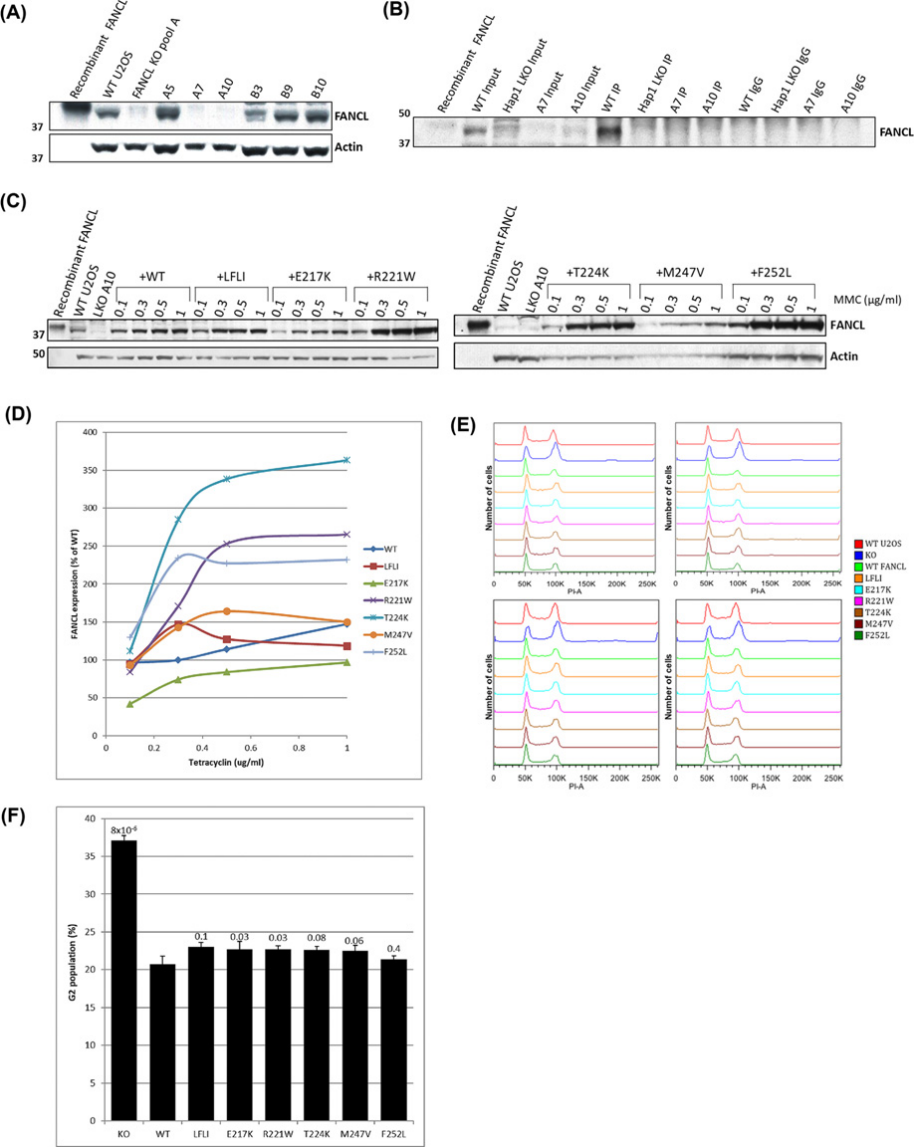
Knockout of FANCL and cellular assay for ICL sensitivity (**A**) Western blot analysis for FANCL from U2OS cell lysate identified two clones (A7 and A10) that were lacking FANCL protein. (**B**) This knockout was confirmed with immunoprecipitation with no visible amounts of FANCL protein in either knockout clone. (**C**) After flipping-in different variants of FANCL, the Tetracycline-inducible expression was assessed by titration. (**D**) The FANCL protein levels from the Tetracycline titration were plotted, and these curves were used to calculate the intercept point for 100% endogenous FANCL expression level. (**E**) FACs analysis shows distribution of cells in G1 and G2 according to DNA content. (**F**) The G2 population percentage is shown for the FANCL knockout (KO) cells, and with the various FANCL proteins expressed. Statistical significance is calculated by *t-*test with *P* values displayed above each bar.

The endogenous copies of FANCL were knocked out using CRISPR-Cas9 gene editing in Human U2OS Trex Flp-In cells (Figure 8A,B). Then, these FANCL knockout cells were transfected with pOG44 recombinase and vectors containing the WT and mutant variants of FANCL within FRT sites. The inducible expression of FANCL mutants with Tetracycline was assessed with concentrations from 0.1 to 1 µg/ml for each cell line (Figure 8C). Titration curves were plotted for expression levels (Figure 8D) so that Tetracycline concentrations could be selected that resulted in expression levels equivalent to endogenous FANCL expression levels.

After 24 h of Tetracycline treatment to induce FANCL expression the cells were treated for 48 h with 7.5 ng/ml of the cross-linking agent MMC. The cells were then fixed in 70% ethanol and stained with propidium iodide and analysed for cell cycle distribution with FACS (Figure 8E,F). FANCL knockout cells show a large G2 accumulation (37.1%), which is rescued by the induced expression of wild-type FANCL (20.7%). All mutants (except F252L) show a statistically significant (*P*≤0.1) difference from the induced WT FANCL cell line. This indicates that these mutations in FANCL cause a defect in the repair of interstrand cross-links.

## Discussion

The critical event in the activation of the FA pathway is the monoubiquitination of the substrates FANCD2 and FANCI, which is catalysed by the E2-E3 pair of Ube2T and FANCL. Mutations have been found in FANCL in non-FA patients’ cancer cells through whole genome sequencing studies. Here, we characterized mutations in the FANCL URD domain to determine whether they affect the function of FANCL, and whether they could be responsible for the progression of cancer in these patients.

The mutations I136V, L154S, W212A and L214A, R221W, R221C, and V287G are found to destabilize the fold of FANCL (Table 1), which would therefore reduce the amount of active FANCL in cancer cells. Of these residues, I136, R221 and V287 appear essential for correct folding of FANCL. Interestingly, the residue R221 is found mutated to Tryptophan in a cancer patient, and to Cysteine in a FA patient. The mutation R221C results in almost entirely unfolded, aggregated protein, explaining why the FA pathway is unable to function in the patient with this mutation, leading to the disease. Similarly, the mutation R221W results in an increase in aggregated protein, a decrease in the melting temperature, and a measured defect in the FA pathway (Table 1). This mutation has a strong negative impact of the function of FANCL, and the sequencing of the cancer cells revealed only 138 other mutations, indicating that the R221W mutation in FANCL could have contributed to the progression of the cancer. The R221 FANCL residue is conserved across species as positively charged (except in *D. melanogaster*) and is located on a loop of the C-terminal URD lobe, directed into the interface between the N- and C-terminal lobes of the URD. It appears R221 could form salt bridges with either D205 or E147 and is essential for the structural integrity and activity of FANCL. We also identified other destabilizing mutants N270K and E289Q; these residues are both within the final two C-terminal helices of the URD domain, and mutations destabilising this region of the domain could contribute to the progression of cancer in patients.

**Table 1:** Summary of FANCL mutations and their effect on protein function

FANCL mutation	Increased aggregation	Decreased melting temperature	Decreased FANCD2 ubiquitination	Decreased FANCD2 pulldown	Increased G2 arrest
L248A, F252A, L254A, I265A			√	√	√
F110S					N.A.
I136V	√	N.A.	N.A.	N.A.	N.A.
L149V				√	N.A.
L154S	√	√		√	N.A.
A192G				√	N.A.
E215Q	√				N.A.
E217K			√		√
R221W	√	√	√	√	√
R221C	√	N.A.	N.A.	N.A.	N.A.
T224K			√		√
M247V	√		√	√	√
F252L			√		
N270K	√			√	N.A.
V287G	√	N.A.	N.A.	N.A.	N.A.
E289Q	√			√	N.A.

The effect of FANCL mutations are summarised in comparison with wild-type FANCL protein, with a tick indicating an observed defect in the properties of FANCL. N.A. (Not Attempted) indicates experiments not performed for these mutant versions of FANCL.

The FANCL hydrophobic patch mutant known to decrease FANCD2 and FANCI binding (L248A, F252A, L254A, I265A) as well as E217K, T224K, and M247V did not destabilize FANCL, but did decrease the activity of FANCL both for the ubiquitination of FANCD2 *in vitro*, and increase cellular sensitivity to the interstrand cross-linking agent MMC (Table 1). L248 and L254 are well conserved, which suggests that these residues may have an important role in recruiting FANCD2, whereas F252 and I265 are less well conserved (Figure 2C), which may be an indication that they are less critical for substrate recognition and ubiquitination. Both FANCL mutations E217K and T224K decreased FA activity without decreasing the binding to FANCD2 (Table 1). This suggests that these residues are important not for substrate binding, but for the catalytic activity of FANCL. Their role may be in interactions with other members of the FA core complex, allosteric regulation of Ube2T [9], or other as yet unknown functions. In the *in vitro* ubiquitination assay E217K decreased the level of Ube2T autoubiquitination, suggesting that this residue is important for stimulating the activity of Ube2T in ubiquitination. It was previously thought that only the RING domain of FANCL was required for the activation of the E2, so this information supports a novel role for the URD domain, expanding its function from substrate binding to catalytic activation.

Overall, the FANCL residues E217, T224 and M247, along with the hydrophobic patch mutant (L248A, F252A, L254A, I265A) have here been shown to be important for FANCL’s E3 ubiquitin ligase activity, and the activation of the FA pathway in cells. These residues highlight the C-terminal lobe of the FANCL URD domain as critical for FANCL’s activity. Furthermore, the recent cryo-EM structure of the FA core complex binding to FANCD2 and FANCI reveals that residues in this region are involved in binding to FANCI [16], with M247 forming part of the interface, underscoring the importance of this domain. The decreased activity of FANCL with these mutations could contribute to the progression of cancer in non-FA patients by decreasing genomic stability.

Ultimately, the present study suggests that mutations in FA genes should be seriously considered as a risk factor for cancer in the general population, as a possible avenue of mutation towards oncogenesis. It will be worth considering that cancer cells with mutations in FA genes will be particularly sensitive to ICL chemotherapeutics, and therefore selective toxicity will be far greater for these treatments making them ideal candidates.

## Abbreviations

C-terminal: Carboxyl-terminus
DTT: Dithiothreitol
*E. coli*: *Escherichia coli*
E2: E2-conjugating enzyme
E3: E3-ligase
ELF: E2-like fold
FA: Fanconi Anemia
FAAP: Fanconi Anemia associated protein
FANC: Fanconi Anemia complementation group
ICL: interstrand cross-link
MMC: Mitomycin C
Ni-NTA: Nickel-nitriloacetic acid
N-terminal: Amino-terminus
PAGE: polyacrylamide gel electrophoresis
PDB: protein data bank
RING: really interesting new gene
SDS: sodium dodecyl sulphate
*T*_m_: melting temperature
tris: tris(hydroxymethyl)aminomethane
URD: UBC-RWD domain
